# Biotechnology, nanotechnology and medicine

**DOI:** 10.1042/ETLS20200350

**Published:** 2020-12-09

**Authors:** Sonia Contera, Jorge Bernardino de la Serna, Teresa D. Tetley

**Affiliations:** 1 Clarendon Laboratory, Physics Department, University of Oxford, Parks Road, Oxford OX1 3PU, U.K.; 2 National Heart and Lung Institute, Imperial College London, London, U.K.

**Keywords:** biotechnology, nanotechnology, translational science

## Abstract

The 1980s mark the starting point of nanotechnology: the capacity to synthesise, manipulate and visualise matter at the nanometre scale. New powers to reach the nanoscale brought us the unprecedented possibility to directly target at the scale of biomolecular interactions, and the motivation to create smart nanostructures that could circumvent the hurdles hindering the success of traditional pharmacological approaches. Forty years on, the progressive integration of bio- and nanotechnologies is starting to produce a transformation of the way we detect, treat and monitor diseases and unresolved medical problems [ 1]. While much of the work remains in research laboratories, the first nano-based treatments, vaccines, drugs, and diagnostic devices, are now receiving approval for commercialisation and clinical use. In this special issue we review recent advances of nanomedical approaches to combat antibiotic resistance, treatment and detection of cancers, targeting neurodegerative diseases, and applications as diverse as dentistry and the treatment of tuberculosis. We also examine the use of advanced smart nanostructured materials in areas such as regenerative medicine, and the controlled release of drugs and treatments. The latter is currently poised to bring ground-breaking changes in immunotherapy: the advent of ‘vaccine implants’ that continuously control and improve immune responses over time. With the increasingly likely prospect of ending the COVID 19 pandemic with the aid of a nanomedicine-based vaccine (both Moderna and BioNTech/Pfizer vaccines are based on lipid nanoparticle formulations), we are witnessing the coming of age of nanomedicine. This makes it more important than ever to concentrate on safety: in parallel to pursuing the benefits of nanomedine, we must strengthen the continuous focus on nanotoxicology and safety regulation of nanomedicines that can deliver the medical revolution that is within our grasp.

## Introduction

The synthesis of novel engineered materials, particularly nanomaterials, has increased rapidly over the last 30 to 40 years; they have a wide range of applications, including engineering, waste disposal, sports goods, electronics, optics, clothing, food, cosmetics — virtually all areas of day-to-day life [2]. This special issue covers a range of applications of novel materials in medicine through the integration of nano- and bio-technology. The unique qualities of materials at the nanometre scale, the ability to manipulate and tailor their physicochemistry at the scale where biomolecular interactions happen opens up a myriad of opportunities in the medical arena, including early detection of biomarkers, specific targeting of cells and tissues, sophisticated drug delivery systems, staging and assessing disease and treatment of degenerative conditions [3]. Of particular interest is the opportunity to address hard-to-treat medical conditions and to discover the mechanisms involved.

Engineered nanomaterials have been defined as having at least one dimension below 100 nm [2,3]. In medicine, this definition is flexible and may include, for example, a nanodrug with particles of 200 nm or more. Furthermore, the use of the term ‘nanoparticle’ can be broad, and include organic (e.g. lipids, biopolymers) and inorganic nanosubstances (e.g. metals, oxides, carbon) that are not spherical, which may be cubes, stars, needles, spheroids, or designed shapes with complex geometries (e.g. in DNA or protein nanotechnology) or other shapes that nevertheless are less than 100 nm aerodynamic diameter. Some of the articles discuss particle properties in the context of the subject, while others do not. The intention is for the reader to appreciate that this term is mostly used in its broadest sense in the following articles.

## Tackling microbial infection

An important area of concern for government agencies and health professionals is the increase in bacterial and fungal resistance to existing therapeutic regimens, and the lack of alternative treatments and strategies to overcome the problem. Add to this overcoming the barriers (e.g. biofilms) that these microbes exhibit/express/display. Many strategies aim to use nanotechnology to tackle this issue.

The conventional use of either oral or intravenous drugs to treat microbial infections is associated with many problems. Existing regimens, including use of large doses to ensure that sufficient quantities reach the targeted organisms, are not always effective and can lead to unwanted side effects and the emergence of drug resistance in the microorganisms. There is a lack of alternative treatments and strategies to overcome this problem; consequently, this is an area of great concern to government agencies and health professionals, and obviously impacts on public health, worldwide. One of the approaches to overcome this problem is the use of nanomaterials to enhance and galvanise the antimicrobial efficacy of existing, and emerging novel, drug therapies.

The article ‘Nano-vehicles to give new lease of life to existing antimicrobials’, by Mela and Kaminski, has focussed on the use of nanomaterials for the purpose of controlled, targeted drug delivery and potentiation of drug activity against bacteria. They discuss how manipulation, specifically of the surface properties, of metallic, organic and DNA-based nanostructures can facilitate functionalisation with the drug(s) and other molecules (such as for cell targeting). Evidence is provided of the efficacy of this strategy in experimental models, with a final focus on the potential of DNA nanostructures (which are designed to create patterns with atomic precision) to deliver antimicrobials.

Some microorganisms develop biofilms, particularly in wounds, containing large numbers of microorganisms, which, apart from the physical barrier, contain numerous other biological ‘barriers’ that confound conventional therapeutic treatment. These obstacles include mediators generated by the microorganisms and the presence of an adverse microenvironment, as well as the presence of a proportion of antibacterial resistant organisms. Joanna Shepherd discusses how nano-sized materials are ideal to breach the biofilm, using a range of materials, which can be honed and modified to break these natural barriers and access the infectious organisms, improving the transfer of the nanomaterials themselves, as well optimising delivery and efficacy of the drug cargo.

## Tuberculosis

Tuberculosis kills millions of people globally; the treatment involves more than one drug formulation over many months and lack of patient compliance can result in multidrug resistance (MDR) of the *Mycobacterial tuberculosis* organism leading to MDR-TB, increased morbidity and mortality. Alzahabi and colleagues explain the background and current treatment of pulmonary TB and the urgent need for new approaches to treating the disease. They make a case for inhalation therapy and with drug-laden nano- and micron-sized materials. They summarise recent research into use of nanomaterials (organic and metallic) as vectors for delivery of existing and novel TB drugs, alone and in combination, to treat TB. They also highlight the use of nanomaterials, such as silver, that confer additional antimicrobial properties to enhance the antimicrobial efficacy of the drug formulation.

## Vaccine implants

Porous polymeric nanostructured implants provide a substrate to deliver drugs and other treatments over time under controlled conditions so avoiding the necessity for repeat administration and providing sustained, controlled release of the active agent. In their article on vaccine implants, Bobbala and Hook report on the advantages of implant reservoirs that enable the slow release of active compounds to enhance interaction with the immune system to generate strong immune defences. They critically appraise different materials currently under investigation to establish vaccine implants for single-shot immunisation that have longevity, are safe and effective. There are exciting opportunities to use this approach to control safer vaccines utilising a variety of combinations of novel and existing antigens, adjuvants and chemokine attractants. This is an exciting, rapidly evolving area of research.

## Dentistry

The use of engineered materials in dentistry is widely understood, particularly for cosmetics purposes. Another important area surrounds dental health and strengthening dental defences. Mok and colleagues review the discipline of nanodentistry, which covers a broad spectrum of the dental practice, including combating infection as well as remineralisation and repair of damaged teeth. Nanotechnology is being applied to all areas of dentistry, where small particle size enhances permeation into lesions, improves therapeutic and cosmetics outcomes, taking advantage of the large surface area to volume ratio of the nanomaterials. Controlled release and site-targeted release of bioactive molecules (e.g. growth factors) and use of drugs at lower doses results in lower side effects whilst achieving desired, optimal outcomes.

## Cancer

Lung cancer is the leading cause of cancer-related deaths and is a difficult-to-treat condition, largely due to late diagnosis. Guinart and colleagues describe the complexities associated with the early detection, diagnosis and treatment of lung cancer and review the diverse applications of gold nanomaterials to address these problems. These range from use as a biosensor for non-invasive diagnosis of the disease, inhalation of functionalised gold particles with targeted drug payload to reduce tumour growth whilst limiting side effects and for photothermal therapy. Ultimately, there is real potential to use gold nanomaterials in theranostics, to diagnose, image and treat the cancer, although such studies are in their infancy and, realistically, some way off translation to the clinic.

## Neurodegenerative disease

The incidence of neurodegenerative conditions such as Alzheimer's and Parkinson's disease is increasing, associated with an aging population and significant cost. Although the mechanisms involved are rapidly unravelling, treatment is hampered by the blood brain barrier (BBB) which filters out and blocks translocation of unwanted blood-born molecules, and consequently many therapeutic and diagnostic agents, to the brain. In an exception to the usual format for Emerging Topics in Life Sciences, an extended length review by Padmanabhan and colleagues describes how nanomaterials are being developed for theranostic purposes in the treatment of Alzheimer's and Parkinson's disease, to enhance the delivery of a range of molecules across the BBB for diagnosis and therapy of these diseases. In addition, to target and modify the behaviour of the BBB itself. They provide detailed descriptions of the *in vitro* and *in vivo* research in this area and the potential for translation into man.

## Tissue repair and regeneration

Mesenchymal stem/stromal cells (MSCs) provide the opportunity to treat degenerative conditions, in the absence of normal tissue repair and replacement. This includes some cardiovascular conditions, fibrosis, osteoarthritis and neurodegenerative disease. However, the infusion of cells does not ensure specific targeting of affected tissues and inadequate engraftment is problematic. Kondo and colleagues review an alternative biotechnological approach, to use MSC-engineered cell sheets that can be generated into 3D structures *in vitro* on thermoresponsive tissue cultureware which allows harvesting of the sheets for transplanting to affected tissues. Therapeutic benefits include induction of paracrine signalling to promote neovascularisation and in the regeneration of cartilage. The article reviews the pre-clinical and clinical evidence supporting the safe use of MSC-containing cell sheets for cartilage repair and tissue regeneration at different tissue sites that exemplifies the utility of this approach for a wide range of medical applications.

## Toxicology

Key to the successful deployment of nanomedicine and to achieve its medical potential is the understanding of the toxicity of nanomaterials. Nanostructures are attractive for medicine, not only because of their chemical and biological activity, but also because they can reach regions that traditional methods cannot: they can be inhaled, ingested and translocate through the skin; inside the body, they can penetrate tissues, cells, and physical barriers (such as moving from the lung to blood circulation or crossing the BBB), and reach vital organs. But their capacity for benefit does not diminish their potential to cause unintended harm. Two decades of nanotoxicology research has shown that the interactions of nanomaterials with cells, animals, humans and the environment are extremely complex [4]. They have been linked to cell apoptosis, inflammation, worsening of asthma, fibrosis, chronic inflammatory lung diseases, and carcinogenesis. Importantly, these toxicological studies have highlighted the physical and functional properties of engineered nanomaterials that should be avoided. Therefore, responsible research and innovation practice in nanomedicine rightly places nanotoxicity and nanotoxicology at the centre of this area of research. Nanotoxicology is also an area of research where scientists can partner with industry, regulators and governments, opening collaborative avenues for scientific research in a multidisciplinary area with multiple stakeholders and contribute to the ongoing debate of regulation and safe use of nanomaterials.

## Summary

- Nanotechnology is a rapidly growing science that offers immense opportunities to target and modify the behaviour of cells and tissues at the nanoscale.
- Integration of bio- and nanotechnologies is transforming the way we detect, treat and monitor disease and thus resolve existing and emerging medical problems.
- Recent advances in nanomedicine are exemplified in this issue, covering a broad range of medical problems — antibiotic resistance, effective vaccines, cancer, tuberculosis, tissue regeneration and dentistry
- However, properties that make nanomaterials so attractive in the medical arena might also cause unintentional harm. Nanotoxicology aims to determine which properties of nanomaterials confer toxicity, and should be avoided, in order to develop safe nanomaterials, particularly for medical purposes.

## Open Access

Open access for this article was enabled by the participation of Imperial College London in an all-inclusive *Read & Publish* pilot with Portland Press and the Biochemical Society under a transformative agreement with JISC.

## Abbreviations

MDR: multidrug resistance
BBB: blood brain barrier
MSCs: mesenchymal stem/stromal cells
